# Flexural Behavior of Two-Span Continuous CFRP RC Beams

**DOI:** 10.3390/ma14226746

**Published:** 2021-11-09

**Authors:** Miao Pang, Sensen Shi, Han Hu, Tiejiong Lou

**Affiliations:** 1 Department of Civil Engineering, Zhejiang University, Hangzhou 310058, China; pm@zju.edu.cn; 2 Hubei Key Laboratory of Roadway Bridge & Structure Engineering, Wuhan University of Technology, Wuhan 430070, China; whutshiss@163.com (S.S.); whuthuh@163.com (H.H.); 3 CEMMPRE, Department of Civil Engineering, University of Coimbra, 3030-788 Coimbra, Portugal

**Keywords:** carbon-reinforced polymer, numerical analysis, structural behavior, moment redistribution

## Abstract

This paper investigates the feasibility of replacing steel bars with carbon-fiber-reinforced polymer (CFRP) bars in continuous reinforced concrete (RC) beams. A numerical model is introduced. Model predictions are compared with the experimental results that are available in the literature. A comprehensive numerical investigation is then performed on two-span CFRP/steel RC beams with *ρ_b_*_2_ = 0.61–3.03% and *ρ_b_*_1_/*ρ_b_*_2_ = 1.5, where *ρ_b_*_1_ and *ρ_b_*_2_ are tensile bar ratios (ratios of tensile bar area to effective cross-sectional area of beams) over positive and negative moment regions, respectively. The study shows that replacing steel bars with CFRP bars greatly improves the crack mode at a low bar ratio. The ultimate load of CFRP RC beams is 89% higher at *ρ_b_*_2_ = 0.61% but 7.2% lower at *ρ_b_*_2_ = 3.03% than that of steel RC beams. In addition, CFRP RC beams exhibit around 13% greater ultimate deflection compared to steel RC beams. The difference of moment redistribution between CFRP and steel RC beams diminishes as *ρ_b_*_2_ increases. ACI 318-19 appears to be conservative, and it leads to more accurate predictions of moment redistribution in CFRP RC beams than that in steel RC beams.

## 1. Introduction

The corrosion of steel bars is responsible for the structural deterioration of reinforced concrete (RC) members around the world, especially those in aggressive environment [1]. Replacing conventional steel bars with non-corrosive fiber-reinforced polymer (FRP) bars can effectively solve the corrosive issue. Apart from their anti-corrosiveness, FRPs have other attractive advantages over steels, such as higher strength, lighter weight and non-magnetism [2]. As a result, FRP materials are widely employed to reinforce/strengthen various concrete members, such as sheets [3,4], bars [5,6] and tendons [7,8]. Nevertheless, FRPs are brittle in nature, and this may result in sudden structural collapse without sufficient warnings. Therefore, a careful assessment on the replacement of steel bars with FRPs in RC elements is essential. A great number of works have been conducted to evaluate the flexural ductility [9,10], deflection [11,12], cracking [13,14] and shear behavior [15] of simply supported FRP RC beams.

Continuous RC beams are preferred in engineering practice, as they have higher stiffness and load-carry capacity than their simply supported counterparts. In the flexural strength design of continuous FRP RC beams, the plastic hinge theory used for continuous steel RC beams is not valid, because of the linear-elastic property of FRP composites. Therefore, ACI 440.1R-06 [2] recommended that no moment redistribution in continuous FRP RC beams be considered. However, redistribution of moments does occur in continuous FRP RC beams during the inelastic range of loading [16,17,18].

Research on continuous FRP RC beams has been performed by different investigators [19,20,21,22]. Among various works, Lou et al. [23] evaluated the moment redistribution against neutral axis depth in carbon and glass FRP (CFRP and GFRP) RC continuous beams, and they also examined various design codes that adopted the neutral axis depth for quantifying the allowable moment redistribution. They concluded that these codes were unsafe in the calculation of moment redistribution in FRP RC beams but the neglect of moment redistribution was over-conservative. In an experimental study, Ashour and Habeeb [24] tested three two-span and two simply supported CFRP RC beams as well as 1 two-span steel RC control beam. The main test variable was the area of CFRP bars. They found that an increase in bottom CFRP bar area resulted in an enhancement in the ultimate load of the beams while the top CFRP bars had negligible influence on the load-carrying capacity of continuous beams. Abushanab et al. [25] tested a total of 10 two-span continuous RC beams with either basalt FRP (BFRP) or steel bars. Their tests showed that BFRP RC beams exhibited larger cracking width, deflection and strain than steel RC beams. In addition, moment redistribution in the beams was considerably improved by increasing the sagging-to-hogging bar ratio or decreasing the stirrup spacing, while the influence of bar ratio was more effective than the influence of stirrup spacing. Basa et al. [26] presented the test results of six GFRP RC continuous beams with the investigated parameters including the sagging-to-hogging bar ratio and the type of GFRP bars. They concluded that in spite of the brittleness of GFRP bars, continuous beams reinforced with GFRP bars exhibited a certain level of ductility, offering sufficient warning prior to collapse.

Although extensive studies have been conducted, behavior of continuous FRP RC beams has not yet been fully addressed. For example, moment redistribution is closely related to the strain in tensile bars, but their relationship in continuous FRP RC beams is yet to be revealed. ACI 318-19 [27] adopted the strain in tensile bars as a single parameter for quantifying the moment redistribution in continuous steel RC beams. Lou et al. [28] proposed a modified ACI 318-19 equation by introducing a new parameter so as to account for the effect of relative stiffness between critical positive and negative moment sections on moment redistribution in continuous steel RC beams. However, the applicability of these approaches to continuous FRP RC beams is yet to be evaluated.

This paper describes an investigation into the feasibility of replacing steel bars with CFRP bars in continuous RC beams. A numerical model developed by Lou et al. [29] is applied. Model predictions are compared with experimental results of CFRP/steel RC continuous beam specimens available in the technical literature. Numerical tests are then performed on two-span continuous CFRP/steel RC beams. Particular attention is placed on the moment redistribution against the strain in tensile bars. Available models for quantifying the moment redistribution based on the bar strain in continuous RC beams are also evaluated.

## 2. Materials and Methods

### 2.1. Materials

The stress–strain relationship for concrete in compression recommended by Eurocode 2 [30] is expressed by the following:

(1) $$\frac{\sigma_{c}}{f_{ck}+8}=\frac{k(\varepsilon_{c}/\varepsilon_{0})-{\left(\varepsilon_{c}/\varepsilon_{0}\right)}^{2}}{1+(k-2)(\varepsilon_{c}/\varepsilon_{0})}$$

where *σ_c_* is the concrete stress; *ε_c_* is the concrete strain; *f_ck_* is the concrete cylinder compressive strength, taken equal to 30 MPa for RC beams used in this numerical investigation; *ε*_0_ is the concrete strain at peak stress; and *k* is a coefficient depending on the concrete grade. The concrete in tension is assumed to be linearly elastic up to cracking, followed by linear tension-stiffening.

CFRP bars are linearly elastic up to rupture. The rupture strength, rupture strain and elastic modulus of CFRP bars used in this numerical investigation are 1450 MPa, 1.09% and 133 GPa, respectively [11].

Steel bars are modeled by a bilinear elastic-hardening law, where the hardening modulus is equal to 1.5% of the elastic modulus. The yield strength and elastic modulus of steel bars used in this numerical investigation are 530 MPa and 200 GPa, respectively.

### 2.2. Finite Element Method

The Timoshenko beam theory has been used for modeling of RC beams [29]. In a two-node Timoshenko beam element, the transverse displacement *w* and rotation *θ* are expressed in terms of the nodal displacements by:

(2) $$w=N_{1}w_{1}+N_{2}w_{2},\;\theta =N_{1}\theta_{1}+N_{2}\theta_{2}$$

where $N_{1}=(l-x)/l$, $N_{2}=x/l$, in which *l* is the element length; the subscripts 1 and 2 represent end nodes. Hence, the curvature, *κ*, and shear strain, *γ*, are expressed by the following:

(3) $$\kappa =-\frac{d\theta }{dx}=B_{b}u^{e},\;\gamma =\frac{dw}{dx}-\theta =B_{s}u^{e}$$

where

(4) $$u^{e}={\left\{\begin{array}{cccc} w_{1} & \theta_{1} & w_{2} & \theta_{2} \end{array}\right\}}^{T}$$

(5) $$B_{b}=\left[\begin{array}{cccc} 0 & -\frac{dN_{1}}{dx} & 0 & -\frac{dN_{2}}{dx} \end{array}\right],\;B_{s}=\left[\begin{array}{cccc} \frac{dN_{1}}{dx} & -N_{1} & \frac{dN_{2}}{dx} & -N_{2} \end{array}\right]$$

Bending moment, *M*, and shear force, *Q*, are then expressed by the following:

(6) $$M=(EI)B_{b}u^{e},\;Q=(GA/k_{s})B_{s}u^{e}$$

where *EI* is the flexural stiffness obtained from the pre-generated moment–curvature relation, *GA* is the shear stiffness and *k_s_* is the shear correction factor.

The principle of virtual work leads to the following equilibrium equations:

(7) $$P^{e}=\int_{l}B_{b}^{T}Mdx+\int_{l}B_{s}^{T}Qdx$$

where

(8) $$P^{e}={\left\{\begin{array}{cccc} Q_{1} & M_{1} & Q_{2} & M_{2} \end{array}\right\}}^{T}$$

Substituting Equation (6) into Equation (7) yields the following element stiffness equations:

(9) $$P^{e}=K^{e}u^{e}=(K_{b}^{e}+K_{s}^{e})u^{e}$$

where

(10) $$K_{b}^{e}=\int_{l}B_{b}^{T}(EI)B_{b}dx,\;K_{s}^{e}=\int_{l}B_{s}^{T}(GA/k_{s})B_{s}dx$$

The stiffness equations for the structure are assembled in the global coordinate system from the contribution of all the elements. After applying proper boundary conditions, the nonlinear equilibrium equations are solved by the incremental–iterative method. The iterative procedure for each increment involves four basic steps, namely formation of the current stiffness matrix, solution of the equilibrium equations, determination of the current state for each element and check of convergence. During the solution process, when any of the constituent materials reaches its ultimate strain capacity, the beam fails and the analysis is therefore terminated. A computer program implementing the aforementioned procedure was written. The program is capable of conducting the full-range nonlinear analysis of continuous steel/FRP RC beams up to failure.

## 3. Comparison of Numerical and Test Data

**Figure 1:**
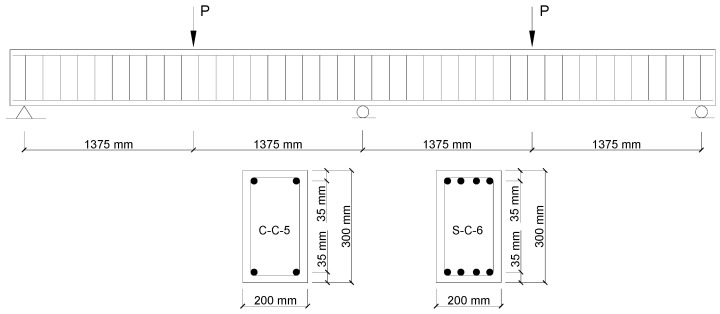
Two-span RC test beams (schematic diagram).

Two continuous RC beams tested by Ashour and Habeeb [24] were selected. One beam was reinforced with CFRP bars (designated as C-C-5), while the other beam was reinforced with steel bars (designated as S-C-6). Both beams were of a rectangular section (200 × 300 mm), continuous over two spans (2750 mm for each span) and subjected to center-point loading symmetrically applied on the spans, as shown in Figure 1. In Beam C-C-5, two CFRP bars with a diameter of 12 mm were provided in both the top and bottom layers of the beam. In Beam S-C-6, four steel bars with a diameter of 12 mm were provided in both the top and bottom layers of the beam. Both specimens were provided with shear reinforcement consisting of steel bars that are 8 mm diameter, with a spacing of 140 mm. The rupture strength, rupture strain and elastic modulus of CFRP bars were 1061 MPa, 0.53% and 200 GPa, respectively. The yield strength and elastic modulus of steel bars were 510.8 MPa and 200 GPa, respectively. The value of *f_ck_* was 28 MPa for Beam C-C-5 and 26.3 MPa for Beam S-C-6.

**Figure 2:**
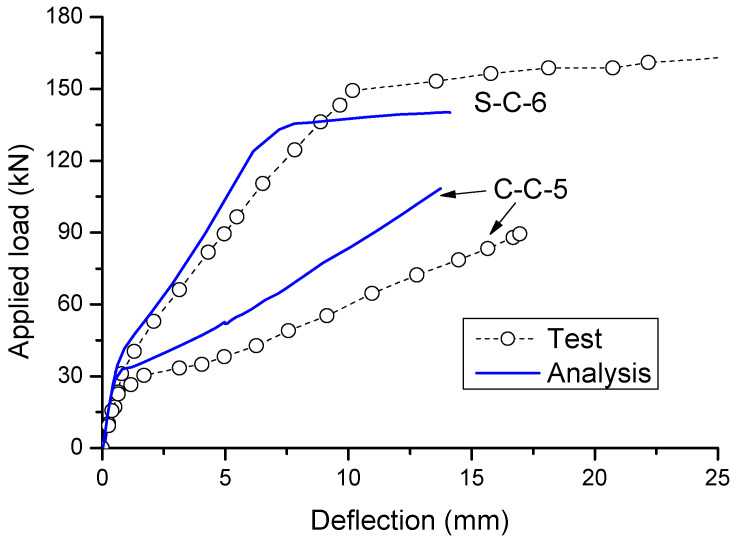
Comparison of numerically obtained load–deflection curves with experimental data.

According to the numerical analysis, crushing failure happens in Beam S-C-6 (i.e., concrete at midspan reaches its ultimate compressive strain of 0.0035), while the rupture failure of CFRP bars takes place in Beam C-C-5 (i.e., CFRP bars at center support reach their rupture strength of 1061 MPa). These failure modes of the specimens are consistent with the experimental observations. The predicted load–deflection behavior of the specimens is compared to the experimental results in Figure 2. It is observed that the post-cracking stiffness by the numerical analysis appears to be higher than the experimental one, while the difference is more apparent for the CFRP RC beam (C-C-5) than for the steel RC beam (S-C-6). This can be explained by the fact that the bond-slip effect, which is more important in CFRP bars than in steel bars, is neglected in the numerical modeling. In addition, the numerical model does not account for the confinement effect in concrete, leading to substantial underestimate in the ultimate deflection in steel RC beams failing by concrete crushing. Despite some discrepancy, the numerical model can capture reasonably the key response characteristics of both specimens (i.e., cracking, yielding and crushing in Beam S-C-6; and cracking and rupture in Beam C-C-5).

## 4. Results and Discussion

**Figure 3:**
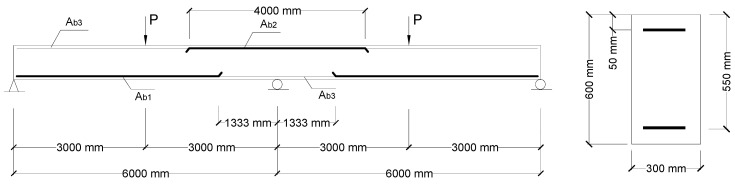
RC continuous beams for numerical evaluation (schematic diagram).

Two-span RC beams under center-point loading symmetrically applied on the spans, as shown Figure 3, were used. The investigated variables are the type of bars (CFRP and steel) and the bar ratio. The arrangement of reinforcing bars is as follows: *ρ_b_*_1_/*ρ_b_*_2_ = 1.5, *ρ_b_*_2_ = 0.61–3.03% and *ρ_b_*_3_ = 0.36%, where *ρ_b_*_1_, *ρ_b_*_2_ and *ρ_b_*_3_ are ratios of tensile bars over positive, negative moment regions and compressive bars, respectively. The bar ratio is defined by *ρ_b_* = *A_b_*/(*bd_b_*), where *A_b_* is the bar area, *b* is the cross-sectional width and *d_b_* is the distance from the centroid of tensile bars to the extreme compressive fiber of the cross-section.

### 4.1. Failure and Cracking Mode

**Figure 4:**
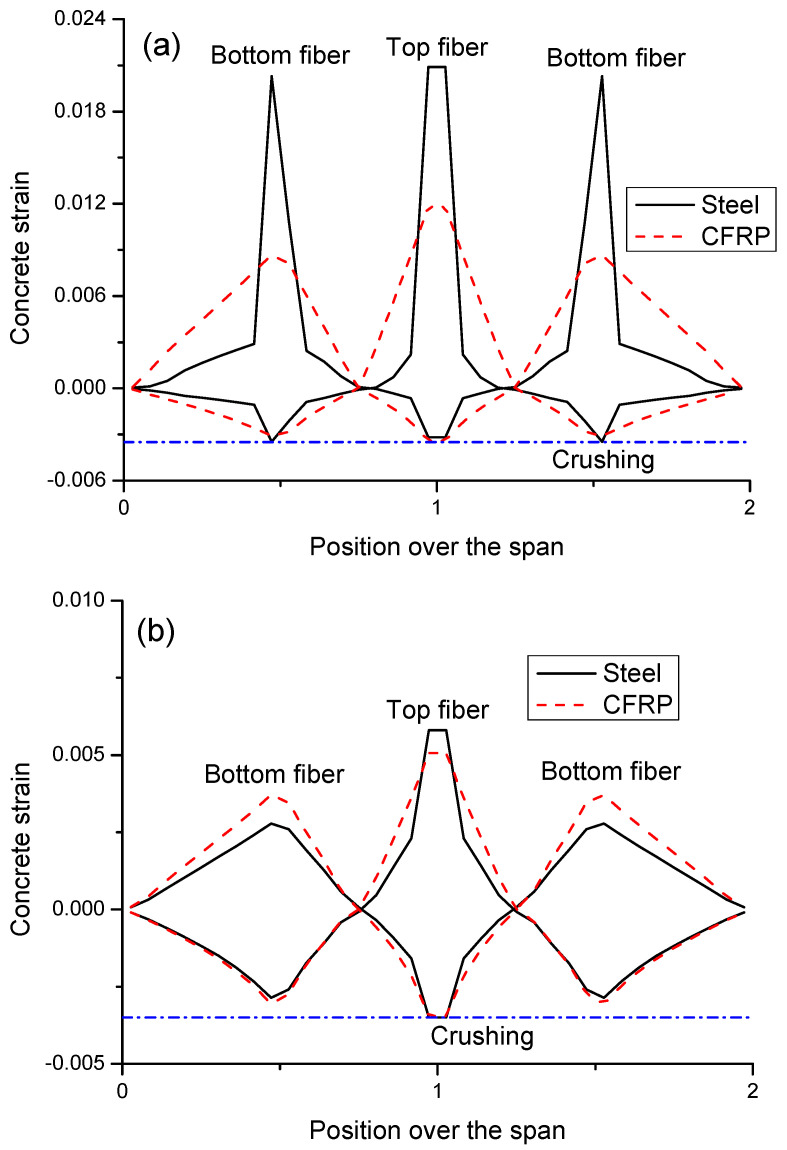
Concrete strain distribution in failure: (**a**) *ρ_b_*_2_ = 0.61%; (**b**) *ρ_b_*_2_ = 2.42%.

Failure of CFRP and steel RC beams occurs when the critical section reaches the ultimate concrete compressive strain of 0.0035 (see Figure 4). For steel RC beams, crushing failure transits from the midspan to the center support when the bar ratio increases from a low to high level. For CFRP RC beams, the beam concrete is crushed at the center support, regardless of the bar ratio. At *ρ_b_*_2_ = 0.61%, CFRP bars are close to their rupture strength when crushing failure happens, indicating an approximately balanced ratio of CFRP bars.

Figure 4a shows that, for steel RC beams with a low *ρ_b_*_2_ level of 0.61%, there appear large concrete tensile strains at the critical sections against relatively small ones over other regions, indicating marked cracking concentration. By using CFRP bars instead of steel bars, the tensile strain distribution or the crack mode is greatly improved, i.e., the crack concentration is relieved and the crack region is more extended. Therefore, it can be concluded that, if concrete beams are lightly reinforced, replacing steel bars with CFRP bars can lead to significant improvement in the crack mode. Figure 4b demonstrates that, at a high *ρ_b_*_2_ level of 2.42%, the maximum tensile strains in the critical sections in both CFRP and steel RC beams are small and the strains over other regions exceed the cracking strain, indicating that small cracks appear all over the beam. CFRP RC beams show a slightly greater crack width at midspan but a slightly smaller crack width at the center support, as compared to steel RC beams.

### 4.2. Moment–Curvature Behavior

**Figure 5:**
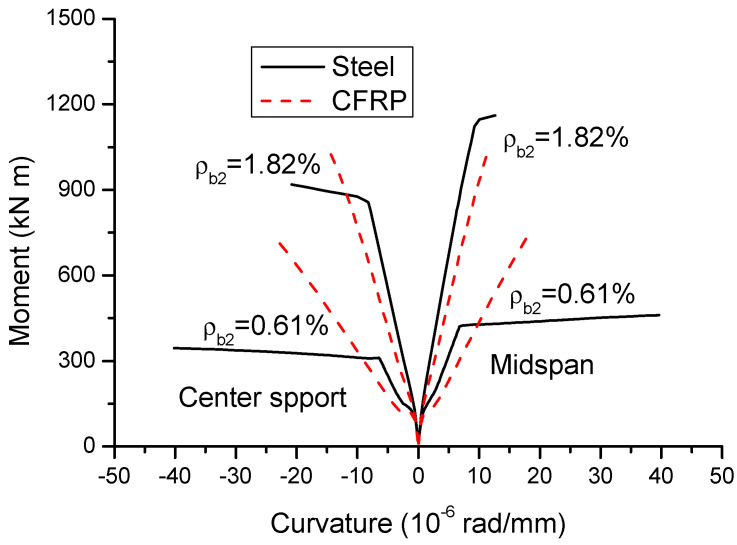
Moment–curvature curves.

Figure 5 shows the moment–curvature curves of CFRP and steel RC beams with *ρ_b_*_2_ = 0.61 and 1.82%. It is clearly demonstrated that steel RC beams exhibit three-stage moment–curvature behavior. Each stage is represented by an approximately linear segment, while each stage transition is featured by a marked reduction in bending stiffness. The first stage, characterized by the elastic behavior, continues up to the occurrence of cracking, followed by the elastically cracked stage until yielding of steel and then by post-yielding stage up to the structural collapse. CFRP RC beams experience only the first and second stages, due to the lack of yielding of FRP bars. Steel and CFRP RC beams behave identically in the first stage, while, in the second stage, steel RC beams show a stiffer behavior than CFRP RC beams attributed to the modulus difference between steel and FRP bars.

**Figure 6:**
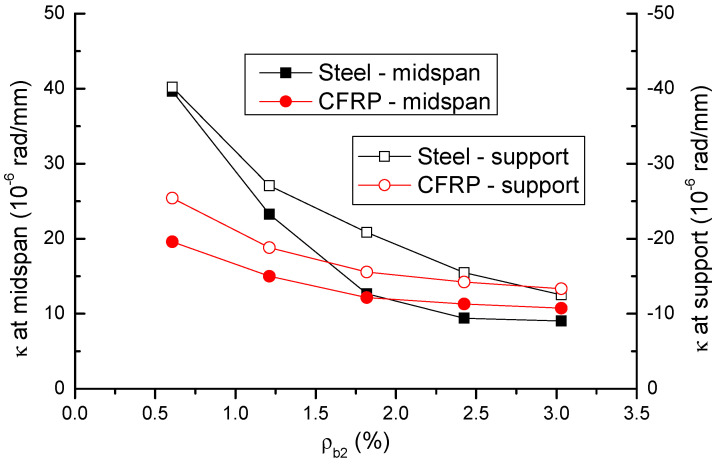
Variation of ultimate curvature with varying bar ratio.

The variation in ultimate curvatures (*κ*) at midspan and center support with varying *ρ_b_*_2_ is shown in Figure 6 and presented in Table 1. At a low *ρ_b_*_2_ level of 0.61%, CFRP bars lead to substantially (i.e., 50.6% at midspan and 36.8% at center support) lower curvature than steel bars. As *ρ_b_*_2_ increases, the decrease in curvatures of CFRP RC beams is slower than that of steel RC beams. As a result, CFRP RC beams exhibit a curvature comparable to that of steel RC beams at high *ρ_b_*_2_ levels of 2.42% and 3.03%.

**Figure 7:**
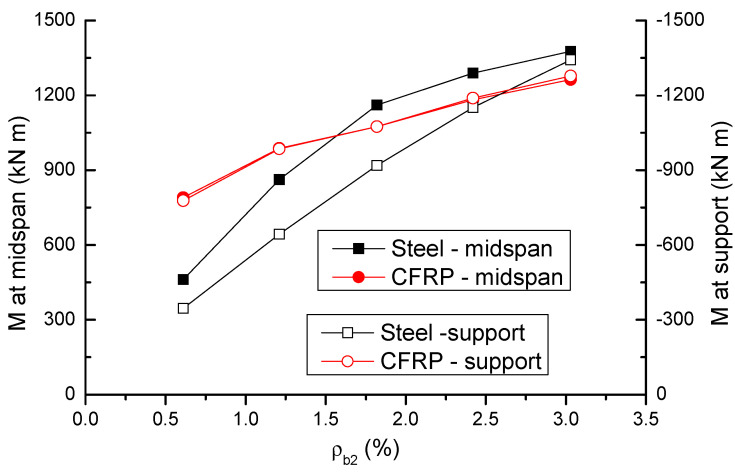
Variation of ultimate moment with varying bar ratio.

The variation in ultimate moments (*M*) at midspan and center support with varying *ρ_b_*_2_ is shown in Figure 7 and presented in Table 1. In steel RC beams, the moment at center support is lower than that at midspan, attributed to the fact that a smaller bar area is provided in the hogging region compared to the sagging region. In CFRP RC beams, however, the moments at midspan and center support are almost identical. This can be explained by the fact that a higher stress in CFRP bars at the center support is achieved in failure, thus compensating the smaller bar area in the section. At a low *ρ_b_*_2_ level of 0.61%, CFRP RC beams show significantly (i.e., 71.5% at midspan and 125.5% at center support) higher moments than steel RC beams. With an increase in *ρ_b_*_2_ level, the increase in moments in CFRP RC beams is slower than that in steel RC beams, attributed to an apparent decrease in stress in CFRP bars. Consequently, CFRP bars may result in lower moments than steel bars at a high *ρ_b_*_2_ level.

### 4.3. Load–Deflection Behavior

**Figure 8:**
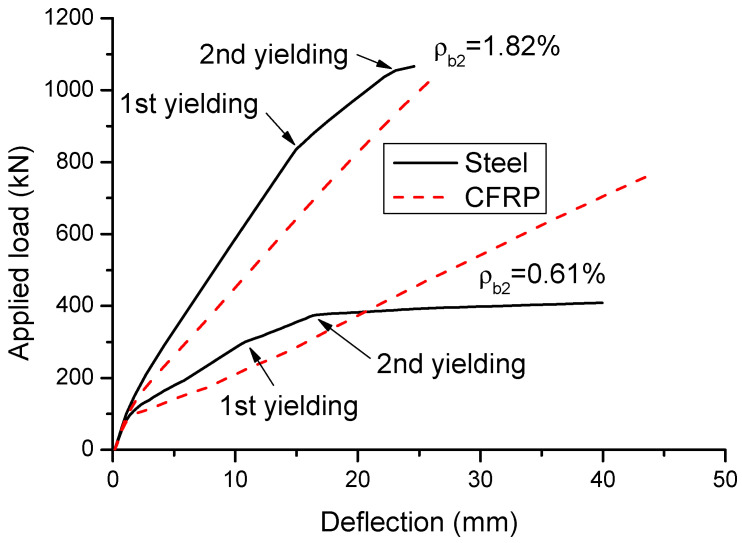
Load–deflection curves.

Figure 8 shows the load–deflection behavior of CFRP and steel RC beams with *ρ_b_*_2_ = 0.6 and 1.82%. Similar to the moment–curvature behavior, the load–deflection curve of CFRP RC beams consists of two stages, namely elastic stage controlled by concrete and post-cracking stage dominated by tensile bars. Each stage is featured by a straight line. For steel RC beams, the first yielding at the center support has limited influences on the load–deflection response, while the second yielding at midspan leads to a remarkable reduction in structural stiffness.

**Figure 9:**
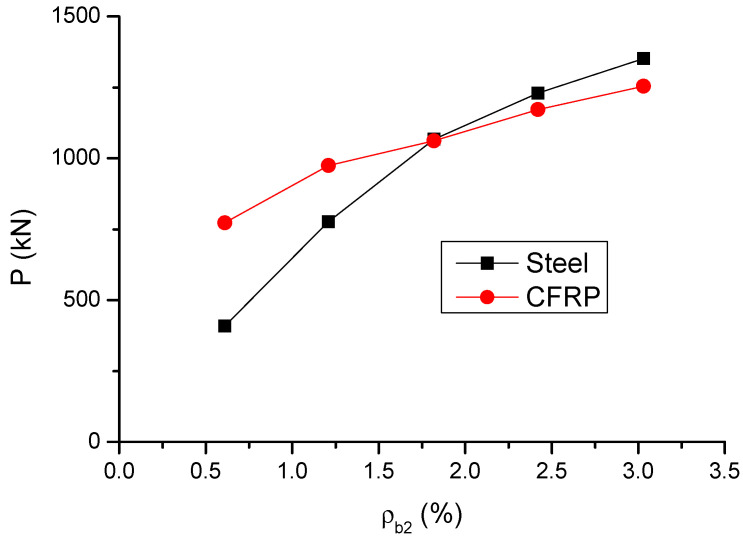
Variation of ultimate load with varying bar ratio.

**Figure 10:**
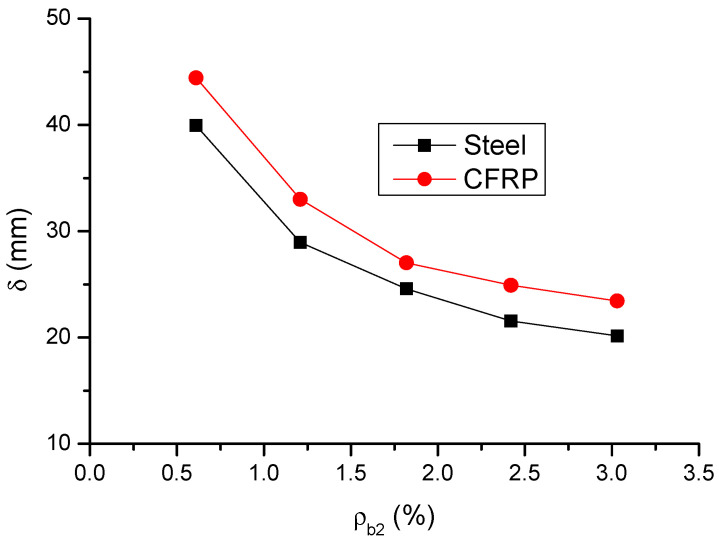
Variation of ultimate deflection with varying bar ratio.

The variation in the ultimate load (*P*) and deflection (*δ*) with varying *ρ_b_*_2_ is shown in Figure 9 and Figure 10, respectively. The results are also presented in Table 1. It is observed that, as *ρ_b_*_2_ increases, the enhancement of the ultimate load of CFRP RC beams is not as apparent as that of steel RC beams. At a low *ρ_b_*_2_ level of 0.61%, the ultimate load of CFRP RC beams is significantly (89%) greater than that of steel RC beams, attributed to that CFRP bars develop substantially higher stress than steel bars. However, at *ρ_b_*_2_ = 3.03%, the ultimate load of CFRP RC beams turns to be 7.2% lower than that of steel RC beams. The ultimate deflection of CFRP RC beams is around 13% greater than that of steel RC beams over the entire *ρ_b_*_2_ levels investigated.

### 4.4. Reaction and Moment Development

**Figure 11:**
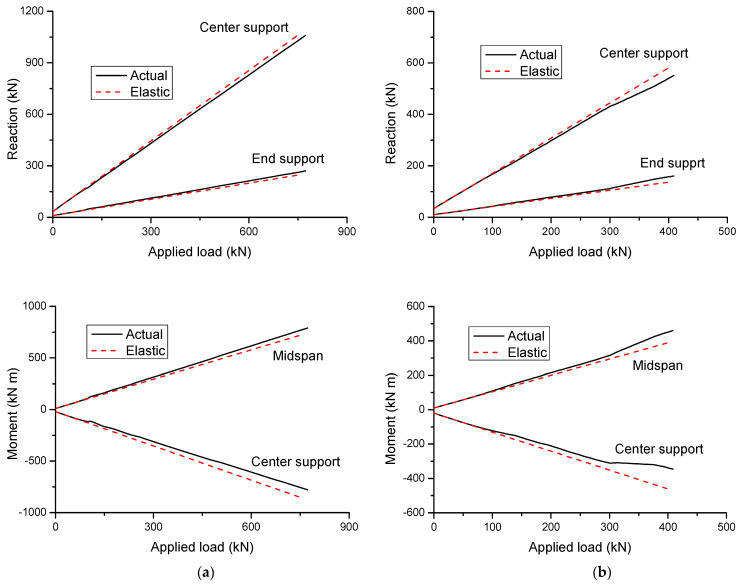
Development of support reactions and bending moments: (**a**) CFRP RC beams and (**b**) steel RC beams.

Figure 11 shows the development of reactions and moments in CFRP and steel RC beams. The results are produced for *ρ_b_*_2_ = 0.61%. The actual values in the graphs are obtained from the nonlinear computer analysis proposed in this study, while the elastic values are obtained based on linear-elastic theory. It can be seen from the figure that the actual values are identical to the elastic ones at the initial loading up to cracking. Thereafter, the actual values begin to deviate from the elastic ones due to redistribution of moments. Because the first cracking appears at the center support, the cracking moments are redistributed from the center support towards the midspan. As a consequence, the reactions grow faster at the end support, and they grow slower at the center support. Correspondingly, the moments increase quicker at midspan and slower at the center support.

In CFRP RC beams, the post-cracking reaction or moment develops linearly with increasing load up to failure, as illustrated in Figure 11a. In addition, over the entire inelastic range of loading, the deviation between actual and elastic reactions or moments is not apparent, indicating a low redistribution of moments in these beams. In steel RC beams, yielding of steel bars may play a critical role in the development of reaction and moment, leading to substantially more deviation between actual and elastic values when compared to CFRP RC beams. Upon the first yielding of steel bars at the center support, further moments are redistributed away from the center support, leading to a further growth of the rate of increase in end support reaction and midspan moment, and correspondingly, a further diminution of the rate of increase in center support reaction and moment, as illustrated in Figure 11b. When steel bars at midspan begin to yield, moments turn to be redistributed from the midspan towards the center support, thereby resulting in a diminution of the rate of increase in the end support reaction and midspan moment, while there is a growth of the increase rate of the center support reaction and moment.

Based on the above discussion, it can be concluded that the developments of support reactions and bending moments, and thereby moment redistribution, are influenced by concrete cracking and steel yielding for steel RC beams, while they are influenced by concrete cracking only for CFRP RC beams.

### 4.5. Moment Redistribution against Bar Strain

Moment redistribution is quantified by the following:

(11) $$\beta =1-M/M_{e}$$

where *β* is the degree of redistribution, *M* is the actual moment and *M_e_* is the elastic moment.

**Figure 12:**
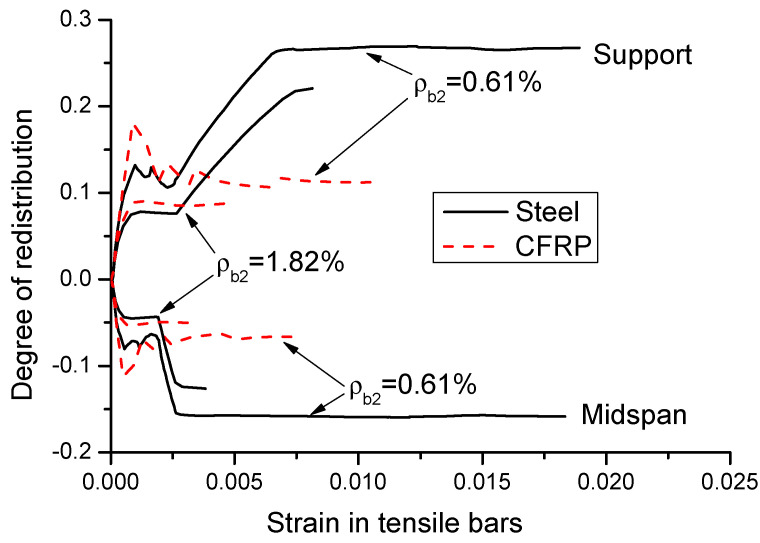
Moment redistribution versus bar strain curves.

Moment redistribution relies strongly on the strain in tensile bars. Figure 12 shows the moment redistribution versus tensile bar strain curves for CFRP and steel RC beams with *ρ_b_*_2_ = 0.61% and 1.82%. Prior to cracking, moment redistribution does not occur (*β* = 0), and the strain in tensile bars is negligible. Moment redistribution takes place after cracking. In general, the curves consist of two distinct stages for CFRP RC beams, while there are two additional stages for steel RC beams. In the first stage, moment redistribution develops linearly with increasing bar strain until the crack evolution stabilizes. In this stage, CFRP RC beams exhibit approximately the same behavior to that of steel RC beams. The second stage is characterized by stabilizing redistribution. For CFRP RC beams, this stage continues until the ultimate failure, accompanied by a substantial variation in bar strain. For steel RC beams, the third stage, triggered by the yielding of steel bars at the center support, is characterized by a quick development of moment redistribution with a limited increase in bar strain. The fourth stage, triggered by yielding of steel bars at midspan, is featured by stabilizing redistribution of moments with varying bar strain up to the ultimate failure.

**Figure 13:**
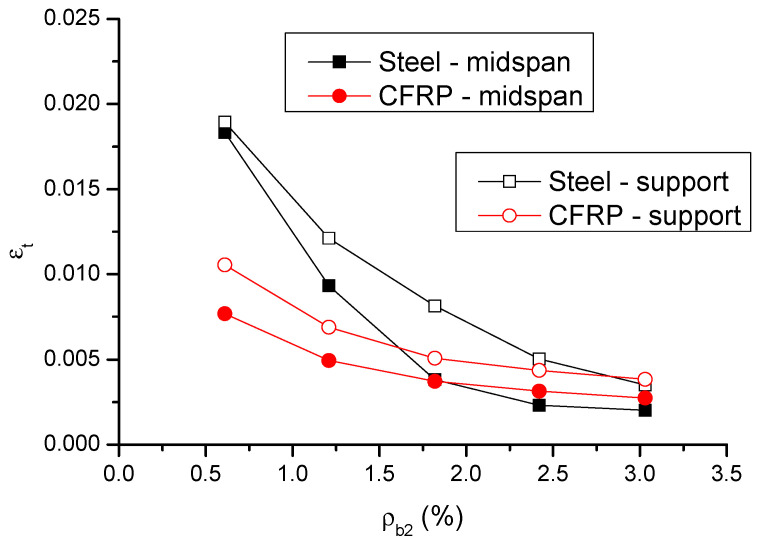
Variation of ultimate bar strain with varying bar ratio.

**Figure 14:**
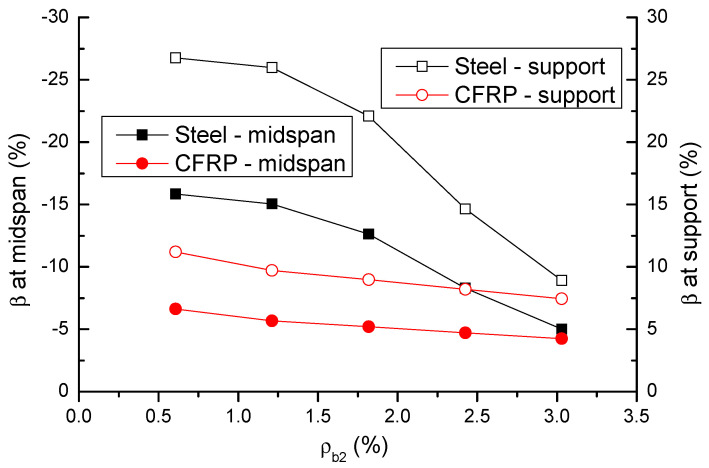
Variation of moment redistribution at ultimate with varying bar ratio.

The change in the strain in tensile bars (*ε_t_*) and moment redistribution (*β*) in failure with the *ρ_b_*_2_ level is displayed in Figure 13 and Figure 14, respectively. The results are also presented in Table 1. It is observed that the bar strain at center support is higher than that at midspan. The strain in CFRP bars is significantly lower than that in steel bars at a low *ρ_b_*_2_ level, but it is close or comparable to that in steel bars at a high *ρ_b_*_2_ level. Moment redistribution at center support is substantially higher than that at midspan. The midspan-to-support-redistribution ratio (absolute value) is around 0.6. Moment redistribution decreases as *ρ_b_*_2_ increases, and this is attributed to the reduction in flexural ductility. The decrease rate of CFRP RC beams is less than that of steel RC beams. CFRP bars lead to significant lower moment redistribution than steel bars at a low *ρ_b_*_2_ level, but the difference narrows as *ρ_b_*_2_ increases. The ratio of moment redistribution in CFRP RC beams to that in steel RC beams varies from 0.42 (at *ρ_b_*_2_ = 0.61%) to 0.85 (at *ρ_b_*_2_ = 3.03%).

## 5. Redistribution Quantification Models based on Bar Strain

When calculating the design moments in continuous RC beams, the current design codes permit an elastic analysis with an adjustment of the elastic moment through the use of *β*. In ACI 318-19 [27], the parameter *ε_t_* is adopted for calculating the permissible redistribution:

(12) $$\beta (\%)=1000\varepsilon_{t}$$

The maximum redistribution is 20%, and *ε_t_* is not less than 0.0075.

**Figure 15:**
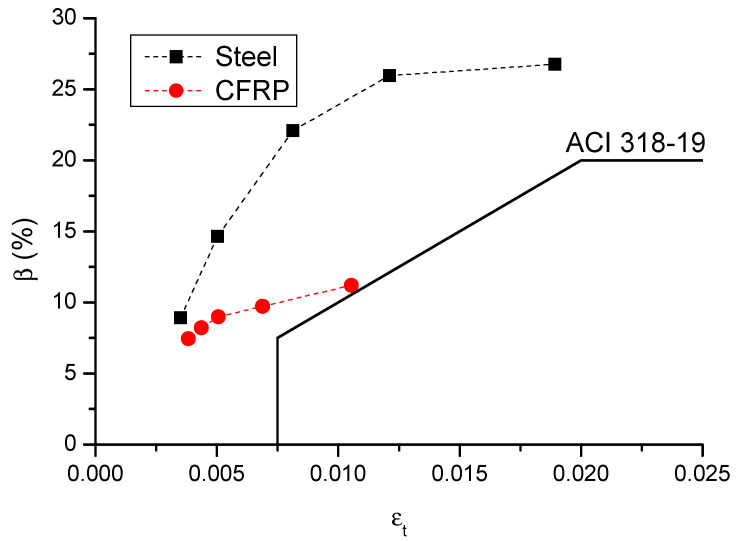
*β*–*ε_t_* relationship.

Figure 15 shows the numerically obtained *β*–*ε_t_* relationship in failure of the center support section of CFRP and steel RC beams, along with the ACI 318-19 curve. The numerical simulations show that, at a given *ε_t_* level, CFRP bars lead to lower moment redistribution than steel bars, and the difference is increasingly notable with the increasing *ε_t_* level. As far as the *β*–*ε_t_* relationship is concerned, ACI 318-19 is consistent with the numerical results, indicating that this code can reflect the tendency of the redistribution variation with the *ε_t_* level. In addition, all of the numerical data are outside of the code curve, suggesting conservative predictions by ACI 318-19.

**Figure 16:**
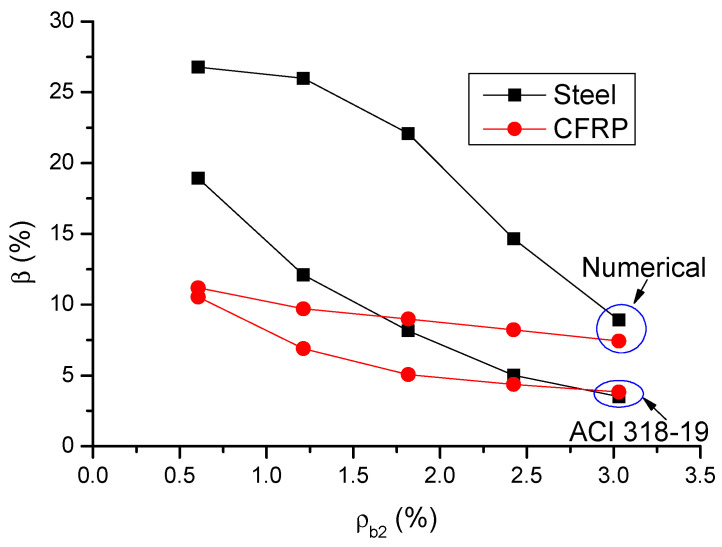
Comparison between numerical and ACI 318-19 predictions regarding the *β*–*ρ_b_*_2_ relationship.

Figure 16 shows a comparison of code and numerical predictions regarding the *β*-*ρ_b_*_2_ relationship for CFRP and steel RC beams. It can be observed that ACI 318-19 can reflect the influence of bar ratio on moment redistribution in RC beams. In addition, according to ACI 318-19, CFRP RC beams generally exhibit lower moment redistribution than steel RC beams; this is especially notable at a low *ρ_b_*_2_ level. This observation is also consistent with the numerical results. Therefore, the effect of bar type on moment redistribution is also reflected in ACI 318-19. From Figure 15 and Figure 16, it is seen that ACI 318-19, which is shown to be conservative, leads to more accurate predictions of moment redistribution in CFRP RC beams than that in steel RC beams.

Lou et al. [28] proposed a modification of Equation (12) by introducing the parameter *ρ_b_*_2_/*ρ_b_*_1_ to predict the moment redistribution in steel RC beams:

(13) $$\beta (\%)=\lambda (1000\varepsilon_{t})$$

(14) $$\lambda =0.68-4.21\ln(\rho_{b2}/\rho_{b1})-2.05\ln^{2}(\rho_{b2}/\rho_{b1})$$

**Figure 17:**
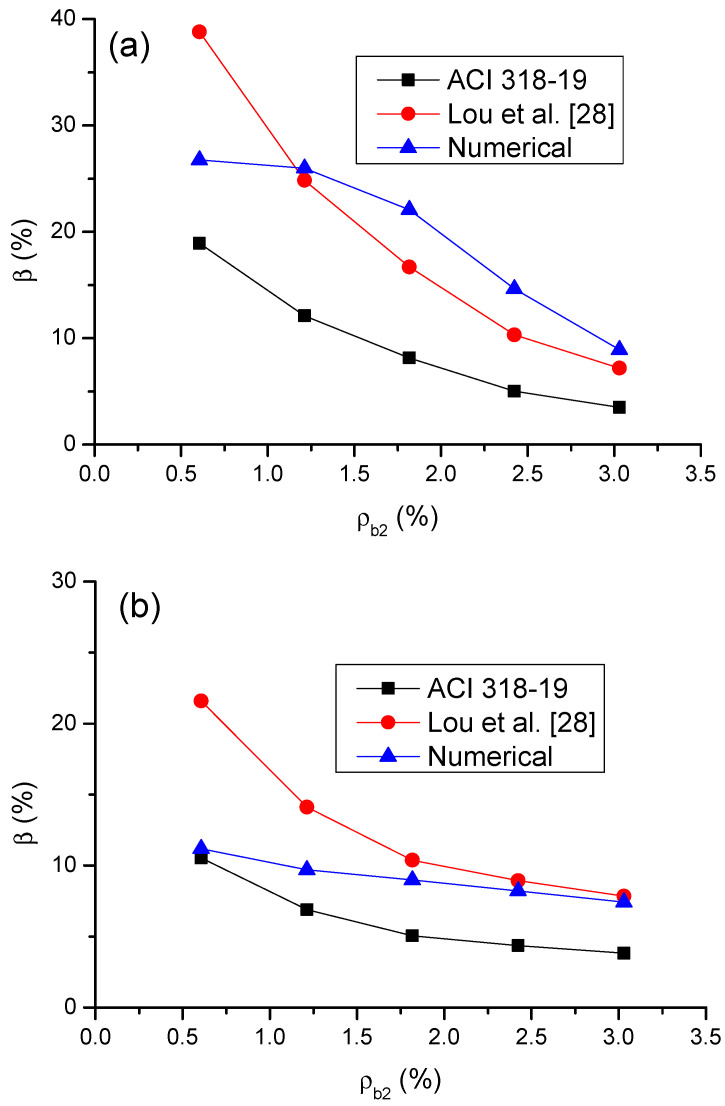
Comparison of the *β*–*ρ_b_*_2_ relationships by simplified equations with numerical simulations: (**a**) steel RC beams and (**b**) CFRP RC beams.

Figure 17 shows a comparison of the predicted moment redistribution by ACI 318-19 and Lou et al. [28] equations with the numerical results. It is seen in Figure 17a that the equation by Lou et al. [28] shows much more accurate predictions in moment redistribution in steel RC beams than the ACI 318-19 equation. However, at a low *ρ_b_*_2_ of 0.61%, the equation by Lou et al. [28] leads to an overestimate when calculating the moment redistribution in steel RC beams. In CFRP RC beams, the equation by Lou et al. [28] is generally unsafe, although it leads to accurate predictions for *ρ_b_*_2_ > 1.82%, as illustrated in Figure 17b. According to numerical simulations, the *β*–*ρ_b_*_2_ relationship for CFRP RC beams is approximately linear. However, according to simplified equations by ACI 318-19 and Lou et al. [28], the relationships exhibit an obviously nonlinear behavior with similar curve shape. This can be explained by the fact that both simplified equations are associated to the parameter *ε_t_*, which varies with varying *ρ_b_*_2_ in a nonlinear manner, as demonstrated in Figure 13.

## 6. Conclusions

A numerical study was conducted to examine the flexural behavior of two-span continuous CFRP RC beams, and the results were compared to those of steel RC beams. The main conclusions of the study are as follows:

- Using CFRP bars instead of steel bars can greatly improve the crack mode of a RC beam with a low bar ratio. While yielding plays a critical role in steel RC beams, the global flexural response (e.g., development of deformations, reactions and moments) of CFRP RC beams is primarily influenced by cracking.
- As *ρ_b_*_2_ increases, the ultimate load of CFRP RC beams increases slower than that of steel RC beams. CFRP RC beams show 89% higher ultimate load at *ρ_b_*_2_ = 0.61% but 7.2% lower ultimate load at *ρ_b_*_2_ = 3.03% than steel RC beams. CFRP RC beams exhibit around 13% greater ultimate deflection than steel RC beams.
- CFRP RC beams show lower moment redistribution due to the lacking of yielding, when compared to steel RC beams. The redistribution difference between CFRP and steel RC beams is notable at a low *ρ_b_*_2_ level and becomes less important with increasing *ρ_b_*_2_.
- ACI 318-19 appears to be conservative and leads to more accurate predictions of moment redistribution in CFRP RC beams than that in steel RC beams. The equation by Lou et al. [28] shows much more accurate predictions of moment redistribution in steel RC beams than ACI 318-19 but is generally unsafe when predicting the moment redistribution in CFRP RC beams.

## Figures and Tables

**Table 1:** Values of typical response characteristics in failure.

Bar	*ρ_b_*_2_ (%)	*P* (kN)	*δ* (mm)	*κ* (10^−6^ rad/mm)		*M* (kN·m)		*ε_t_* (‰)		*β* (%)	
				Mid	Sup	Mid	Sup	Mid	Sup	Mid	Sup
Steel	0.61	408.64	39.94	39.65	−40.19	460.69	−345.05	18.33	18.92	−15.85	26.76
	1.21	775.98	28.95	23.25	−27.08	862.60	−643.24	9.33	12.11	−15.05	25.97
	1.82	1066.75	24.59	12.68	−20.84	1161.10	−918.56	3.82	8.14	−12.63	22.09
	2.42	1229.71	21.54	9.39	−15.49	1289.30	−1151.03	2.31	5.02	−8.30	14.65
	3.03	1351.61	20.14	9.05	−12.51	1376.39	−1342.54	2.01	3.50	−5.01	8.92
CFRP	0.61	772.69	44.42	19.60	−25.39	790.22	−778.15	7.68	10.54	−6.62	11.20
	1.21	973.99	33.01	14.99	−18.82	988.29	−985.88	4.94	6.89	−5.67	9.71
	1.82	1060.95	27.03	12.16	−15.54	1074.22	−1074.91	3.71	5.07	−5.19	8.98
	2.42	1171.36	24.91	11.30	−14.21	1182.73	−1189.13	3.14	4.36	−4.71	8.21
	3.03	1254.32	23.43	10.75	−13.33	1262.98	−1277.50	2.73	3.83	−4.24	7.44

Note: Mid = midspan; Sup = center support.

## Data Availability

The data presented in this study are available on request from the corresponding author.
